# Complement C5a and Clinical Markers as Predictors of COVID-19 Disease Severity and Mortality in a Multi-Ethnic Population

**DOI:** 10.3389/fimmu.2021.707159

**Published:** 2021-12-13

**Authors:** Farhan S. Cyprian, Muhammad Suleman, Ibrahim Abdelhafez, Asmma Doudin, Ibn Mohammed Masud Danjuma, Fayaz Ahmad Mir, Aijaz Parray, Zohaib Yousaf, Mohammed Yaseen Ahmed Siddiqui, Alaaedin Abdelmajid, Mohammad Mulhim, Shaikha Al-Shokri, Mohammad Abukhattab, Ranad Shaheen, Eyad Elkord, Abdul Latif Al-khal, Abdel-Naser Elzouki, Guillermina Girardi

**Affiliations:** ^1^ Biomedical and Pharmaceutical Research Unit, QU Health, Qatar University, Doha, Qatar; ^2^ Department of Basic Medical Sciences, College of Medicine, Member of QU Health, Qatar University, Doha, Qatar; ^3^ Institute of Microbiology, University of Veterinary and Animal Sciences, Lahore, Pakistan; ^4^ Department of Math and Science, Community College of Qatar, Doha, Qatar; ^5^ Internal Medicine Department, Hamad General Hospital, Hamad Medical Corporation, Doha, Qatar; ^6^ Qatar Metabolic Institute, Academic Health System, Hamad Medical Corporation, Doha, Qatar; ^7^ The Neuroscience Institute, Academic Health System, Hamad Medical Corporation, Doha, Qatar; ^8^ Communicable Diseases Center, Hamad Medical Corporation, Doha, Qatar; ^9^ Natural and Medical Sciences Research Centre, University of Nizwa, Nizwa, Oman; ^10^ Biomedical Research Centre, School of Science, Engineering and Environment, University of Salford, Manchester, United Kingdom

**Keywords:** COVID-19, SARS-CoV-2, C5a anaphylatoxin, blood indices, biomarkers

## Abstract

Coronavirus disease-2019 (COVID-19) was declared as a pandemic by WHO in March 2020. SARS-CoV-2 causes a wide range of illness from asymptomatic to life-threatening. There is an essential need to identify biomarkers to predict disease severity and mortality during the earlier stages of the disease, aiding treatment and allocation of resources to improve survival. The aim of this study was to identify at the time of SARS-COV-2 infection patients at high risk of developing severe disease associated with low survival using blood parameters, including inflammation and coagulation mediators, vital signs, and pre-existing comorbidities. This cohort included 89 multi-ethnic COVID-19 patients recruited between July 14^th^ and October 20^th^ 2020 in Doha, Qatar. According to clinical severity, patients were grouped into severe (n=33), mild (n=33) and asymptomatic (n=23). Common routine tests such as complete blood count (CBC), glucose, electrolytes, liver and kidney function parameters and markers of inflammation, thrombosis and endothelial dysfunction including complement component split product C5a, Interleukin-6, ferritin and C-reactive protein were measured at the time COVID-19 infection was confirmed. Correlation tests suggest that C5a is a predictive marker of disease severity and mortality, in addition to 40 biological and physiological parameters that were found statistically significant between survivors and non-survivors. Survival analysis showed that high C5a levels, hypoalbuminemia, lymphopenia, elevated procalcitonin, neutrophilic leukocytosis, acute anemia along with increased acute kidney and hepatocellular injury markers were associated with a higher risk of death in COVID-19 patients. Altogether, we created a prognostic classification model, the CAL model (C5a, Albumin, and Lymphocyte count) to predict severity with significant accuracy. Stratification of patients using the CAL model could help in the identification of patients likely to develop severe symptoms in advance so that treatments can be targeted accordingly.

## Introduction

Severe acute respiratory syndrome coronavirus 2 (SARS-CoV-2), first reported as a novel pneumonia in Wuhan (China), has resulted in above 119 million infections and approximately 2.5 million deaths globally (1). SARS-CoV-2 is one of seven coronaviruses that are capable of infecting humans. Three of these viruses can cause severe disease, namely SARS-CoV, MERS-CoV and SARS-CoV-2, the other four HKU1, NL63, OC43 and 229E are linked to mild disease symptoms (2). Generally, coronaviruses are known to cause respiratory, enteric, hepatic, and neurological symptoms in their host, with a wide spectrum of disease severity (3). COVID-19 patients have a variable presentation of symptoms including fever, non-productive cough, dyspnea, myalgia, fatigue and pneumonia accompanied with normal or reduced leukocyte counts (4). Thus, COVID-19 disease symptoms range from asymptomatic and mild phenotype, to the severe infection leading to acute respiratory distress syndrome (ARDS) and multiorgan failure with poor survival rates (5). The mortality rate varies among populations and patient demographics and is reported to be higher within the elderly, individuals with pre-existing comorbidities, and immunocompromised patients (6).

In addition, a role for the cytokine storm has been suggested as a crucial player in determining disease severity, including the development of pulmonary intravascular coagulation. Among the mechanisms contributing to multiorgan failure, an interplay between inflammation and coagulation has been shown critical in COVID-19 patients (7). Exacerbated coagulation, characterized by elevated levels of D-dimer (>1 μg/ml) were observed in COVID-19 patients, and an association with poor prognosis was reported (8). Moreover, the presence of a prothrombotic state in multiple organs is supported by autopsies of COVID-19 patients independent of disease time-course (9). Particularly, megakaryocytes and platelet-rich thrombi are found in the lungs, heart, and kidneys (10). Remarkably, COVID-19-associated thrombosis is linked with increased morbidity and mortality in critical cases (11–13). Accumulating evidence suggests a correlation between increased level of inflammatory mediators, including cytokines and chemokines, and COVID-19 disease progression. For instance, SARS-CoV-2 infection severity is linked to high blood levels of C-reactive protein, ferritin, and D-dimers (14, 15). The clinical symptoms documented in the severe form of COVID-19 resemble those observed in the cytokine release syndrome (CRS) (16–18). CRS is characterized by a robust release of pro-inflammatory cytokines such as IL-6 (19). A significant correlation between levels of IL-6 and an increased risk of death was observed in COVID-19 patients (17). Hence, tocilizumab and sarilumab were proposed as promising therapeutic strategies to reduce mortality in COVID-19, by blocking IL-6 signalling, and ameliorating the deleterious effects of the inflammatory storm (20). The RECOVERY trial study recently demonstrated the effectiveness of tocilizumab and dexamethasone combined in improving the survival rate and reducing the risk for ventilatory assistance in COVID-19 patients (21).

Furthermore, the complement system, an important arm of innate immunity, has been proposed as a crucial mediator in lung inflammation in SARS-CoV-2 infection. Generally, the complement system triggers a cascade, upon its activation by specific recognition pathways, leading to the generation of cleavage products that opsonize and eliminate pathogens, regulating inflammatory responses, and coordinating adaptive immunity (REF). Indeed, an exaggerated complement activation induced by excessive stimuli such as viruses, may have a damaging effect by turning the complement system into a pathogenic effector in numerous diseases, especially thrombosis and sepsis (22). An active cross-talk between the complement system and the coagulation cascade has been established (23) (24). Interestingly, increased levels of complement split product C5a were detected in the plasma and bronchoalveolar lavage fluid (BALF) of COVID-19 patients (25). In addition, a higher expression level of C5aR1 receptors were measured in pulmonary myeloid cells in the blood of SARS-CoV-2 infected patients, supporting a role for the C5a-C5a receptor interaction in the pathophysiology of ARDS (23, 25). In this study, several serological and biological variables were measured in patients with SARS-CoV-2 infection and their association with disease severity was analysed. We tested the hypothesis that C5a is a novel potential candidate to predict COVID-19 disease severity. Furthermore, we tested the association between C5a and pro-inflammatory and coagulation biomarkers in COVID-19 patients. The results from this study might aid the identification of patients at risk of developing severe COVID-19 disease or mortality, and in discerning pharmacological interventions to improve patient outcomes.

## Materials and Methods

### Study Approval

This study was conducted in accordance with the Code of Ethics of the World Medical Association. Ethics committee approval was obtained from the Medical Research Center at Hamad Medical Corporation (MRC-05-084, Immunological and immune-genetic investigations in COVID-19 patients with varying disease severity, 06/21/2020). All the patients gave their informed consent to participate.

### Study Design and Data Collection

This prospective cohort study included 89 randomly selected patients, diagnosed with COVID-19 in Doha between July 14^th^ and October 20^th^, 2020. Selection criteria for participants was age (between 35 and 65 years), a positive SARS-CoV-2 PCR test result (a CT value < 30) and residency in Qatar. Upper respiratory tract specimens (throat and nasopharyngeal swabs) were tested for SARS-CoV-2 infection using TaqPath COVID-19 Combo Kit (Thermo Fisher Scientific, Waltham, Massachusetts), or Cobas SARS-CoV-2 Test (Roche Diagnostics, Rotkreuz, Switzerland). All consented patients were recruited form Hazem Mebairek Hospital, Qatar’s centre of communicable disease control (CDC) and Um Gharan quarantine facility. SARS-CoV-2 testing is routinely offered to all individuals presenting with symptoms suggestive of COVID-19, those who had close contact with confirmed cases, and all returning travellers.

Participants were stratified into three categories namely severe, mild and asymptomatic. Patients were categorized in the severe group based on requirement of oxygen support and ICU admission (n=33). While mild cases were categorized based on clinical symptoms and positive radiographic findings indicating pulmonary involvement (n=33). COVID-19 patients with mild to severe disease (n=66) were hospitalized for inpatient management, out of which 33 (50%) were admitted to ICU. In the severe group, 23 patients (70%) needed mechanical ventilation, of which fourteen patients (42%) died with respiratory failure listed as the primary cause of death. Standard of care for hospitalized patients consisted of supportive care and antiviral therapy, with individual regimens selected based on severity of disease, the presence of comorbidities, contra-indications and potential drug-drug interactions. The patients with a positive SARS-CoV-2 PCR and no longitudinal clinical presentation were labelled as asymptomatic cases (n=23). Blood samples were collected at the time of diagnosis, prior to isolation, or hospitalization. Clinical and laboratory investigations including diastolic blood pressure, body mass index (BMI), viral load, number of comorbidities, routine blood tests including complete blood cell counts were performed. In addition, the blood levels of electrolytes, glucose, albumin, total protein, C-reactive protein, procalcitonin, IL-6, D-dimers, ferritin, urea, and liver enzymes were determined. Complement activation C5a was measured in plasma using Human C5a/Complement C5a ELISA Kit (Sigma, St Lousi, MO, USA). Survival data including whether the patients were still alive or not as the end of survey date was obtained from the CERNER electronic healthcare system. This study was performed in accordance with the Reporting of Observational Studies in Epidemiology (STROBE) recommendations (26).

### External Validation of Existing Severity and Mortality Models in COVID-19 Patients

Nowadays, various early prediction models have emerged amid the COVID-19 pandemic, aiming at optimizing patient stratification and reducing morbidity and mortality (27). In this study, we systematically tested several existing mortality and severity models, based on simplicity and ease of use. The tested mortality models included the CURB-65 (confusion, urea, respiratory rate, BUN, age>65) (28), the CRB-65 (confusion, respiratory rate, BUN, age>65) (29), the pneumonia severity index (PSI) (30), and the ANDC score (age, NLR, D-dimer, CRP) (31). The first three models have been validated and extensively used to predict 30-day mortality in community-acquired pneumonia, whereas the ANDC has only recently been employed to predict COVID-19 mortality. These scoring systems need rigorous external validation, through testing in different populations. Therefore, we further attempted to validate four COVID‐19 severity models, including the CALL (comorbidity, age, lymphocyte count, lactate dehydrogenase), CALL‐interleukin‐6 (IL‐6) scores, Haifeng et al. model (lymphocyte count and albumin), and Zhenyu et al. model (age, albumin, comorbidity, CRP) (32).

### Accuracy Assessment of Prediction Models

Accuracy of COVID severity and mortality models was assessed using the area under the receiver-operator characteristic curve (AUC). For internal validation of the accuracy estimates and to reduce overfit bias, we used 1000 bootstrap resamples. The area under the curve (AUC) was calculated to detect the model with the best discriminatory capacity. A model with AUROC>0.8 is known to be of excellent discriminatory ability (31). In addition, we tested model calibration using the “rms” package.

### Statistical Analysis

All statistical analyses were conducted with R statistical software (version 4.0.4, R Foundation). Shapiro-Wilk test was used to examine covariates normality and choose an appropriate statistical test. Since all variables had a non-normal distribution, comparisons between different groups of severity and survival were performed using the Mann-Whitney test for continuous variables, and Fisher’s Exact Test for categorical. Values were reported as medians and interquartile range [IQR]. Two tailed p-values were calculated and *p*-value <0.05 was considered statistically significant. Spearman rank correlation tests were used to assess the correlation between different blood parameters. The Kaplan-Meier method was used to plot survival curves.

## Results

### Clinical Characterization of COVID-19 Patients Upon Diagnosis

In order to find a set of early predictors of severity and mortality, we analysed the clinical history, instrumental variables and laboratory tests of 89 patients diagnosed with COVID-19, aged between 35 and 65 years [median, IQR 48 (42-56)]. This multi-ethnic study included patients from thirteen nationalities: Qatar, Pakistan, Bangladesh, India, Siri Lanka, Philippines, Nepal, Egypt, Libya, Tunisia, Yemen, Eretria, and US. In agreement with previously published studies of COVID-19 cohorts, patients with comorbidities, including diabetes, hypertension, dyslipidemia, cardiovascular disease, renal disease, liver disease and/or asthma experienced more severe symptoms, requiring admission to the intensive care unit (ICU) (33). In particular, 70% out of 33 severe cases required mechanical ventilation. Within the severe group, 36% had at least 1 comorbidity, 44% and 39% had at least 2 and 3 comorbidities respectively (**Table 1**). Furthermore, at the time of diagnosis, a low diastolic blood pressure and a high respiratory rate were observed in severe/critical cases that required ICU admission compared to patients with mild and asymptomatic patients (**Supplementary Figure 1A**). Interestingly, out of 69 patients with available BMI data, 71% were overweight [27.9 (24.7, 30.4)] (BMI>25 kg/m^2^) including asymptomatic [26.1 (23.8, 30.1)], mild [28.8 (25.8, 31.7)], and severe [27.3 (24.5, 29.4)] (**Supplementary Figure 1B** and **Table 1**). The nutritional status of our cohort according to the WHO guidelines was 26% normal weight, 46% pre-obesity, 19% obesity class I, 4% obesity class II, and 3% obesity class III (**Table 1**) (34, 35). It is noteworthy to mention that hyperglycemia was observed in COVID-19 patients with mild and severe disease, despite insulin treatment (**Table 1**). While some studies suggest a direct association between viral load and COVID-19 disease severity, the viral load in our cohort did not show a difference between asymptomatic, mild or severe cases at the time of diagnosis (**Supplementary Figure 1C**) (36–38).

**Table 1:** Demographic, clinical and laboratory measurements of asymptomatic, mild, and severe COVID-19.

Characteristic	N	Total N = 89^1^	Asymptomatic N = 23^1^	Mild N = 33^1^	Severe N = 33^1^	*p*-value^2^
**Survival [%]**	89		23 (31%)	33 (44%)	19 (25%)	**<0.001**
**Gender (Male) [%]**	89		21 (26%)	29 (36%)	31 (38%)	0.9
**At least 1 Comorbidity**	89		17 (31%)	18 (33%)	20 (36%)	0.3
**At least 2 Comorbidities**	89		7 (22%)	11 (34%)	14 (44%)	0.6
**At least 3 Comorbidities**	89		5 (28%)	6 (33%)	7 (39%)	>0.9
**Hospitilization duration**	89	7 (0-32)	0 (0-0)	6 (0-10)	46 (29-86)	**<0.001**
**Age [years]**	89	48 (42-56)	43 (38-48)	47 (43-55)	54 (46-59)	**0.002**
**COVID-19 Average CT**	78	25.6 (18.7-29.5)	25.4 (19.2-28.2)	26.2 (18.5-31.7)	24.4 (19.3-28.0)	0.5
**Diastolic blood pressure [mmHg]**	89	75 (64-83)	78 (71-90)	76 (67-82)	68 (57-80)	**0.019**
**Body Mass Index (BMI) [kg/m^2^]**	68	28.1 (24.8-30.4)	27.6 (23.8-30.4)	28.8 (25.8-31.7)	27.3 (24.5-29.4)	0.4
**Nutritional status**	68					0.4
Normal weight			4 (22%)	5 (28%)	9 (50%)	
Obesity class I			3 (23%)	6 (46%)	4 (31%)	
Obesity class II			0 (0%)	1 (33%)	2 (67%)	
Obesity class III			0 (0%)	2 (100%)	0 (0%)	
Pre-obesity			2 (6.5%)	12 (39%)	17 (55%)	
Underweight			0 (0%)	0 (0%)	1 (100%)	
**Glucose [mmol/L]**	89	6.3 (5.3-8.8)	5.5 (4.8-6.4)	6.0 (5.3-8.2)	7.3 (5.9-10.3)	**0.005**
**White blood cell count (WBC) [x10^3^/µL]**	89	7.1 (4.9-10.3)	6.3 (5.2-7.5)	5.9 (4.2-7.5)	11.2 (8.0-13.6)	**<0.001**
**Lymphocyte count [x10^3^/µL]**	89	1.40 (1.00-1.90)	1.70 (1.60-2.35)	1.30 (1.00-1.90)	1.00 (0.60-1.70)	**0.001**
**Absolute neutrophil count (ANC) [x10^3^/µL]**	89	4.6 (3.1-7.8)	3.7 (2.5-4.7)	3.4 (2.4-5.8)	8.3 (6.3-11.5)	**<0.001**
**Neutrophil-to-lymphocyte ratio**	89	2.8 (2.0-8.7)	2.1 (1.9-2.5)	2.4 (1.5-3.9)	9.3 (3.6-17.7)	**<0.001**
**Monocyte count[x10^3^/µL]**	89	0.60 (0.40-0.80)	0.60 (0.50-0.80)	0.40 (0.30-0.70)	0.70 (0.40-0.80)	0.10
**Eosinophil count[x10^3^/µL]**	89	0.00 (0.00-0.10)	0.10 (0.00-0.20)	0.00 (0.00-0.10)	0.00 (0.00-0.10)	0.066
**Basophil count[x10^3^/µL]**	89	0.030 (0.010-0.040)	0.030 (0.020-0.030)	0.020 (0.010-0.040)	0.040 (0.010-0.050)	0.2
**Lymphocyte [%]**	89	23 (9-30)	28 (25-30)	27 (18-35)	8 (5-19)	**<0.001**
**Neutrophil [%]**	89	68 (58-83)	59 (54-64)	66 (53-74)	84 (73-90)	**<0.001**
**Monocyte [%]**	89	7.7 (5.2-9.3)	8.8 (7.4-11.7)	7.9 (6.0-9.5)	5.4 (3.4-8.7)	**<0.001**
**Eosinophil [%]**	89	0.70 (0.00-1.90)	1.80 (0.35-3.10)	0.50 (0.00-1.50)	0.30 (0.00-0.90)	**0.019**
**Basophil [%]**	89	0.30 (0.20-0.50)	0.50 (0.35-0.50)	0.30 (0.20-0.60)	0.30 (0.10-0.50)	**0.019**
**Red blood cell count (RBC) [x10^6^/µL]**	89	4.70 (3.80-5.30)	5.10 (4.80-5.40)	5.10 (4.70-5.40)	3.40 (3.00-4.10)	**<0.001**
**Hematocrit (Hct) [%]**	89	40 (33-44)	44 (42-46)	41 (39-44)	31 (27-36)	**<0.001**
**Hemoglobin (Hgb) [g/dL]**	89	13.00 (10.40-14.70)	14.60 (13.80-15.40)	13.70 (12.90-14.70)	9.60 (7.90-11.50)	**<0.001**
**Hemoglobin A1C (HbA1C) [%]**	47	6.20 (5.60-7.55)	5.65 (5.40-6.00)	6.90 (5.75-8.85)	6.20 (5.70-7.00)	0.2
**Ferritin [µg/L]**	62	661 (286-1,151)	396 (181-582)	404 (220-786)	1,131 (536-1,634)	**<0.001**
**Mean corpuscular volume (MCV) [fL]**	89	86 (82-91)	86 (83-89)	83 (78-87)	90 (86-93)	**<0.001**
**Mean cell hemoglobin (MCH) [pg]**	89	28.60 (26.70-30.00)	28.80 (27.55-30.40)	27.90 (25.80-29.60)	29.20 (27.80-30.00)	0.087
**Mean corpuscular hemoglobin concentration (MCHC) [g/dL]**	89	33.10 (31.60-33.90)	33.60 (32.25-33.95)	33.60 (32.50-34.20)	32.30 (31.10-33.30)	**0.033**
**Red blood cell distribution width (RDW-CV) [%]**	89	13.50 (12.50-15.10)	12.30 (12.00-13.15)	13.30 (12.50-13.80)	15.20 (14.10-17.30)	**<0.001**
**Platelet [× 10^9^/L]**	85	246 (194-326)	248 (228-298)	216 (190-306)	269 (190-344)	0.8
**Mean platelet volume (MPV) [fl]**	86	10.55 (9.70-11.38)	10.60 (9.70-11.20)	10.30 (9.70-10.90)	10.85 (10.10-11.90)	0.2
**Platelet distribution width (PDW) [fl]**	56	13.5 (11.5-15.7)	15.3 (14.4-15.6)	13.8 (11.0-15.7)	12.6 (11.6-15.8)	0.7
**Prothrombin time (PT) [second]**	55	12.10 (11.35-13.20)	12.35 (11.80-13.02)	11.40 (11.05-11.70)	12.55 (11.95-13.88)	**0.002**
**International Normalized Ratio (INR)**	55	1.00 (1.00-1.10)	1.05 (1.00-1.12)	1.00 (1.00-1.00)	1.10 (1.00-1.20)	0.12
**D-Dimer [mg/L FEU]**	64	0.87 (0.37-2.40)	0.30 (0.26-0.38)	0.43 (0.30-0.57)	2.18 (1.20-4.73)	**<0.001**
**Fibrinogen [g/L]**	40	4.15 (3.00-5.67)	26.30 (16.55-29.65)	4.60 (3.90-5.90)	3.80 (2.98-4.95)	**0.021**
**Partial thromboplastin time (APTT) [second]**	55	31 (28-37)	30 (26-33)	31 (28-33)	33 (29-40)	0.2
**Complement component 5a (C5a) [pg/ml]**	89	1,383 (929-1,786)	1,009 (722-1,265)	1,289 (749-1,687)	1,815 (1,407-2,400)	**<0.001**
**C-reactive protein (CRP) [mg/L]**	89	16 (5-58)	4 (1-14)	21 (7-83)	27 (6-77)	**<0.001**
**Interleukin-6 (IL-6) [pg/mL]**	52	32 (12-57)	14 (8-19)	32 (13-52)	34 (8-82)	0.4
**High-sensitivity Troponin-T [ng/mL]**	40	26 (8-108)	7 (6-88)	8 (5-10)	36 (24-150)	**<0.001**
**Lactic acid [mmol/L]**	35	1.70 (1.10-2.20)	1.80 (1.12-2.52)	1.37 (1.10-1.85)	1.80 (1.30-2.30)	0.5
**Uric acid [µmol/L]**	51	305 (266-384)	326 (277-406)	313 (274-380)	300 (148-376)	0.5
**Bicarbonate [mmol/L]**	89	25.0 (23.0-28.0)	26.0 (25.0-27.0)	24.9 (23.0-26.0)	26.0 (23.0-32.0)	**0.034**
**Sodium [mmol/L]**	89	138.0 (136.0-141.0)	138.0 (136.0-140.0)	137.0 (135.0-139.0)	141.0 (137.0-147.0)	**0.006**
**Potassium [mmol/L]**	85	4.40 (4.00-4.70)	4.60 (4.12-5.10)	4.15 (4.00-4.55)	4.40 (4.00-4.70)	0.3
**Chloride [mmol/L]**	89	102.0 (99.0-104.0)	102.0 (99.0-103.0)	101.0 (99.0-103.0)	104.0 (98.0-109.0)	0.079
**Phosphorus [mmol/L]**	46	1.14 (1.00-1.31)	1.30 (1.28-1.32)	1.14 (1.01-1.16)	1.14 (0.98-1.42)	0.4
**Magnesium [mmol/L]**	60	0.89 (0.83-0.96)	0.87 (0.84-0.90)	0.87 (0.79-0.92)	0.94 (0.87-1.01)	**0.030**
**Total Protein [g/L]**	80	71 (64-77)	77 (70-80)	72 (68-75)	67 (57-72)	**<0.001**
**Albumin [g/L]**	89	32 (27-39)	41 (38-44)	35 (32-39)	26 (23-29)	**<0.001**
**Bilirubin [mg/dL]**	80	8 (5-13)	8 (4-10)	8 (6-12)	8 (6-16)	0.4
**Urea [mmol/L]**	89	5 (4-10)	4 (3-5)	4 (3-5)	13 (6-21)	**<0.001**
**Creatinine [µmol/L]**	89	78 (63-92)	81 (72-88)	78 (66-92)	66 (54-98)	0.5
**Creatine kinase (CK) [U/L]**	45	103 (58-245)	98 (84-114)	73 (61-133)	133 (57-359)	0.7
**Alkaline phosphatase (ALP) [U/L]**	80	88 (63-110)	82 (69-92)	86 (62-100)	99 (73-166)	0.14
**Alanine aminotransferase (ALT) [U/L]**	77	31 (20-64)	25 (18-36)	35 (24-72)	34 (22-76)	0.085
**Aspartate aminotransferase (AST) [U/L]**	72	28 (23-50)	26 (18-29)	27 (22-49)	36 (25-54)	0.10
**Lactate dehydrogenase (LDH) [U/L]**	53	347 (249-462)	318 (250-386)	249 (214-339)	429 (336-623)	**<0.001**
**Low-density lipoprotein (LDL) [mmol/L]**	16	2.85 (2.28-3.70)	2.00 (1.60-2.40)	2.90 (2.41-4.07)	3.19 (2.77-3.43)	0.4
**Triglyceride [mmol/L]**	52	1.80 (1.35-2.62)	1.10 (0.95-2.15)	1.36 (1.20-1.77)	2.20 (1.70-3.05)	**<0.001**
**Vitamin D [ng/mL]**	56	18 (13-23)	22 (14-24)	18 (14-21)	17 (12-24)	0.7
**Calcium [mmol/L]**	89	2.25 (2.14-2.35)	2.34 (2.28-2.42)	2.26 (2.15-2.34)	2.16 (2.06-2.25)	**<0.001**
**Adjusted calcium [mmol/L]**	89	2.39 (2.30-2.46)	2.31 (2.26-2.37)	2.35 (2.29-2.46)	2.45 (2.39-2.53)	**<0.001**
**Procalcitonin [ng/mL]**	47	0.27 (0.10-0.55)	0.03 (0.03-0.14)	0.16 (0.05-0.30)	0.44 (0.14-0.83)	**0.007**

^1^Statistics presented: n (%); Median (25%-75%).

^2^Statistical tests performed: Fisher’s exact test; chi-square test of independence; Kruskal-Wallis test.

Bold p-values are those below the threshold of 0.05.

### Changes in Complete Blood Count Are Associated With Disease Severity and Mortality in SARS-CoV-2 Infected Patients

**Figure 1:**
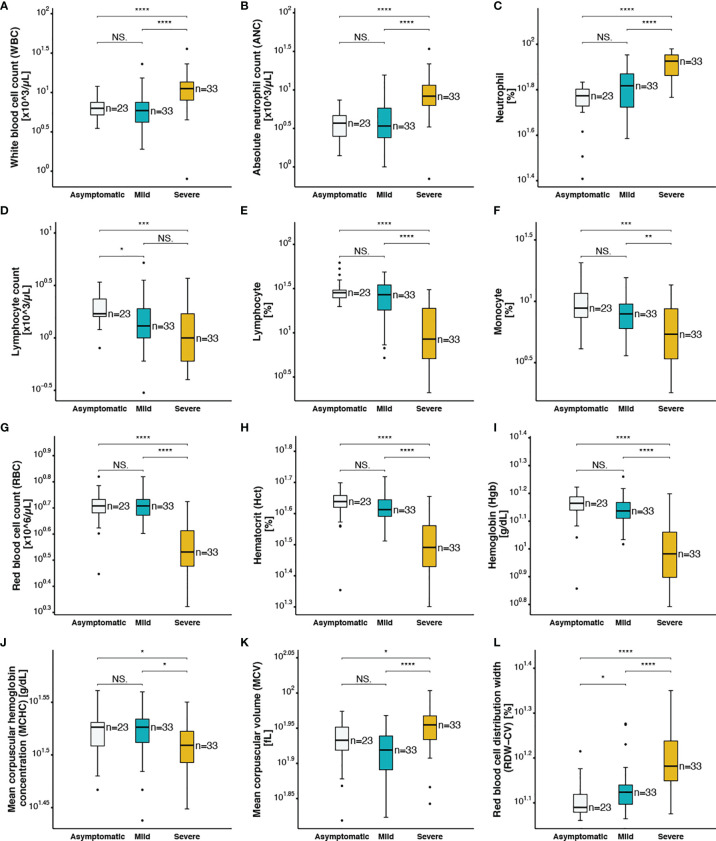
Complete blood count parameters of asymptomatic, mild and severe COVID-19 patients **(A)** white blood cell count, **(B)** absolute neutrophil count, **(C)** neutrophil (%), **(D)** lymphocyte count, **(E)** lymphocyte (%), **(F)** monocyte (%), **(G)** red blood cell count, **(H)** hematocrit, **(I)** hemoglobin, **(J)** mean corpuscular hemoglobin concentration, **(K)** mean corpuscular volume, **(L)** red blood cell distribution width. The following symbols were used to indicate statistical significance: NS, p > 0.05; *p ≤ 0.05; **p ≤ 0.01; ***p ≤ 0.001; ****p ≤ 0.0001.

The association between hematological and serological biomarkers changes and disease severity in SARS-CoV-2 infection was analysed (**Table 1**). The analysis of the CBC variables using Wilcoxon rank sum test showed a significant increase in white blood cell count (WBC) (**Figure 1A**), absolute neutrophil count (ANC) (**Figure 1B**), percentage of neutrophils (**Figure 1C**), and neutrophil-to-lymphocyte ratio (**Figure 3I**) in severe patients compared to mild and asymptomatic cases. In contrast, lymphocyte count (**Figure 1D**), percentage of lymphocytes (**Figure 1E**), and monocytes (**Figure 1F**) showed significantly lower levels in severe cases compared to non-severe counterparts. On the other hand, the total monocyte counts, as well as eosinophil and basophil percentages were not different among the three groups with varying disease severity (**Table 1**). In addition, changes in red blood cell parameters were observed among patients with different COVID-19 disease severity. Decreased red blood cell count (RBC) (**Figure 1G**), haematocrit (Hct) (**Figure 1H**), haemoglobin (Hgb) levels (**Figure 1I**), and mean corpuscular hemoglobin concentration (MCHC) (**Figure 1J**) were detected in severe patients compared to mild and asymptomatic cases. On the other hand, mean corpuscular volume (**Figure 1K**) and red blood cell distribution width (RDW-CV) (**Figure 1L**) were elevated in severe cases compared to patients without symptoms and with mild symptoms. Moreover, compared to patients who survived, non-survivors had significantly higher levels of total WBC (**Supplementary Figure 2A**), ANC (**Supplementary Figure 2B**), neutrophils (%) (**Supplementary Figure 2C**), mean corpuscular volume **(**MCV) (**Supplementary Figure 2D**), ferritin (**Supplementary Figure 2E**), and RDW-CV (**Supplementary Figure 2F**). However, patients who did not survive despite medical intervention showed significantly lower levels of lymphocyte count (**Supplementary Figure 2G**), lymphocyte (%) (**Supplementary Figure 2H**), monocyte (%) (**Supplementary Figure 2I**), RBC (**Supplementary Figure 2J**), Hct (%) (**Supplementary Figure 2K**), and Hgb (**Supplementary Figure 2L**).

### COVID-19 Severity Is Associated With Increased Levels of C5a, Coagulation, and Inflammation Markers

**Figure 2:**
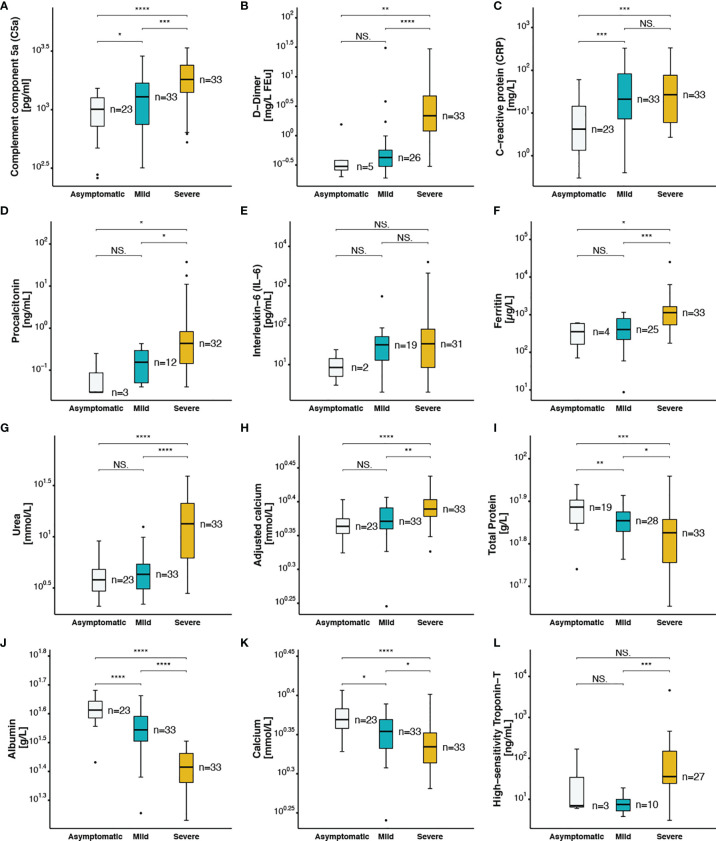
Coagulation and inflammatory markers in asymptomatic, mild and severe COVID-19 patients **(A)** complement component 5a, **(B)** D−Dimer, **(C)** C−reactive protein, **(D)** procalcitonin, **(E)** Interleukin−6, **(F)** ferritin, **(G)** urea, **(H)** adjusted calcium, **(I)** total protein, **(J)** albumin, **(K)** calcium, **(L)** high−sensitivity Troponin−T. The following symbols were used to indicate statistical significance: NS, p > 0.05; *p ≤ 0.05; **p ≤ 0.01; ***p ≤ 0.001; ****p ≤ 0.0001.

The plasma concentration of complement split product C5a was measured using ELISA in all 89 COVID-19 patients. Although, the normal plasma level of C5a is less than 120 pg/ml, higher levels were detected in all samples (>260 pg/ml), including the asymptomatic patients. Interestingly, C5a plasma levels increased proportionally to COVID-19 disease severity (p<0.001) (**Figure 2A**). Prothrombin time (p=0.002) was higher in severe patients, whereas fibrinogen was lower in the severe group compared to non-severe patients (p=0.021) (**Table 1**). Regarding survival outcomes, median circulating C5a levels were further compared between survivors [1,289 (775-1,690)] and non-survivors [1,670 (1,375-2,191)] and were found statistically significant (p=0.035) (**Supplementary Figure 3A**). A procoagulant state characterized by platelet activation, increased mean platelet volume (p<0.001) (**Supplementary Figure 3B**), platelet distribution width (p=0.010) (**Supplementary Figure 3C**), and partial thromboplastin time (p=0.006) (**Supplementary Figure 3D**) was observed in patients with severe disease. Furthermore, within the severe group the higher values were observed in patients that did not survive. Elevated D-dimer levels were also observed in the severe group (**Figure 2B**) and particularly in patients who did not survive [4.15 (1.49-5.67)] (p<0.001) (**Supplementary Figure 3E**) as compared to mild and asymptomatic cases.

Recent studies have shown an association between higher levels of inflammatory markers and SARS-CoV-2 infection severity (7). In this line, increased peripheral leukocytes and neutrophils numbers, and higher levels of C-reactive protein (CRP) were observed in patients with severe disease [27 (6-77)] compared to mild [21 (7-83)] and asymptomatic patients [4 (1-14)] (p<0.001) (**Figure 2C**). Procalcitonin, another acute-phase protein usually below the limit of detection in clinical assays in the blood of healthy individuals, was found in high amounts in patients with severe disease [0.44 (0.14-0.83)] compared to patients with no symptoms [0.03 (0.03-0.14)] and to those with mild clinical presentation [0.16 (0.05-0.30)] (p=0.007) (**Figure 2D**). Provocatively, higher levels of CRP (**Supplementary Figure 3F**) and procalcitonin (**Supplementary Figure 3G**) were detected in patients that did not survive compared to those who survived, with a prominent increase in CRP levels [74 (21-158) vs 13 (5-40)] (p=0.004). A mildly elevated but statistically non-significant elevation of IL-6 levels was observed in severely ill patients when compared to other groups (**Figure 2E**). Another key acute-phase reactant that we investigated was ferritin, whereas an increase in ferritin levels protects the host by limiting the free iron needed for pathogen growth and survival, it can also play a pro-inflammatory role, contributing to the cytokine storm (39). Within the group of critically ill COVID-19 patients, hyperferritinemia (> 500 µg/L) was observed with a median of 1,131 [536-1,634] vs 396 [181-582] and 404 [220-786] in patients with no or mild symptoms respectively (**Figure 2F**). Accumulated evidence is in support of a higher mortality rate of patients with cardiovascular diseases as a result of SARS-CoV-2 infection (40). In this line higher levels of high-sensitivity cardiac troponin T (HS-TnT) as a marker of disease progression, was determined in severe cases compared to mild patients (**Figure 2L**). Particularly, higher levels were observed in patients that did not survive (**Supplementary Figure 3M**).

### SARS-CoV-2 Disease Severity Is Associated With Changes in Kidney and Liver Function Parameters

Acute kidney injury has been reported in adult patients following SARS-CoV-2 infection with multifactorial causality including cytokine storm, hypoxia, increased coagulation and impaired glomerular filtration. Interestingly, we observed significantly higher levels of urea in severe patients compared to asymptomatic and mild patients (p<0.001) (**Figure 2G**). High urea levels indicative of impaired renal function were observed in non-survivors (**Supplementary Figure 3I**). To further investigate the association between renal function and COVID-19 disease severity and mortality, electrolytes were measured. Patients with severe complications of COVID-19 presented with hypernatremia (**Table 1**) and hypocalcemia (**Figure 2K**), in which low calcium levels correlated with increased mortality (**Supplementary Figure 3L**). Recently, COVID-19 patients were classified as severe or non-severe based on total protein levels in the serum (41). In our study, we found that circulating total protein (**Figure 2I**) and albumin levels (**Figure 2J**) were inversely correlated with disease severity. As a result, calcium values adjusted for the albumin concentration were highest in the severe COVID-19 cases (**Figure 2H**). The levels of total protein (**Supplementary Figure 3J**), albumin (**Supplementary Figure 3K**), and calcium (**Supplementary Figure 3L**), were significantly lower in patients who died compared to those who survived.

Involvement of different organs is another hallmark of COVID-19 severity. In the current cohort, significantly elevated levels of liver enzymes, alkaline phosphatase (ALP) (**Supplementary Figure 3N**), alanine aminotransferase (ALT) (**Supplementary Figure 3O**), aspartate aminotransferase (AST) (**Supplementary Figure 3P**), and lactate dehydrogenase (LDH) (**Supplementary Table 1**) were measured in non-survivors compared to survivors.

### C5a Is Correlated With Several Blood Indices in SARS-CoV2 Infection

**Figure 3:**
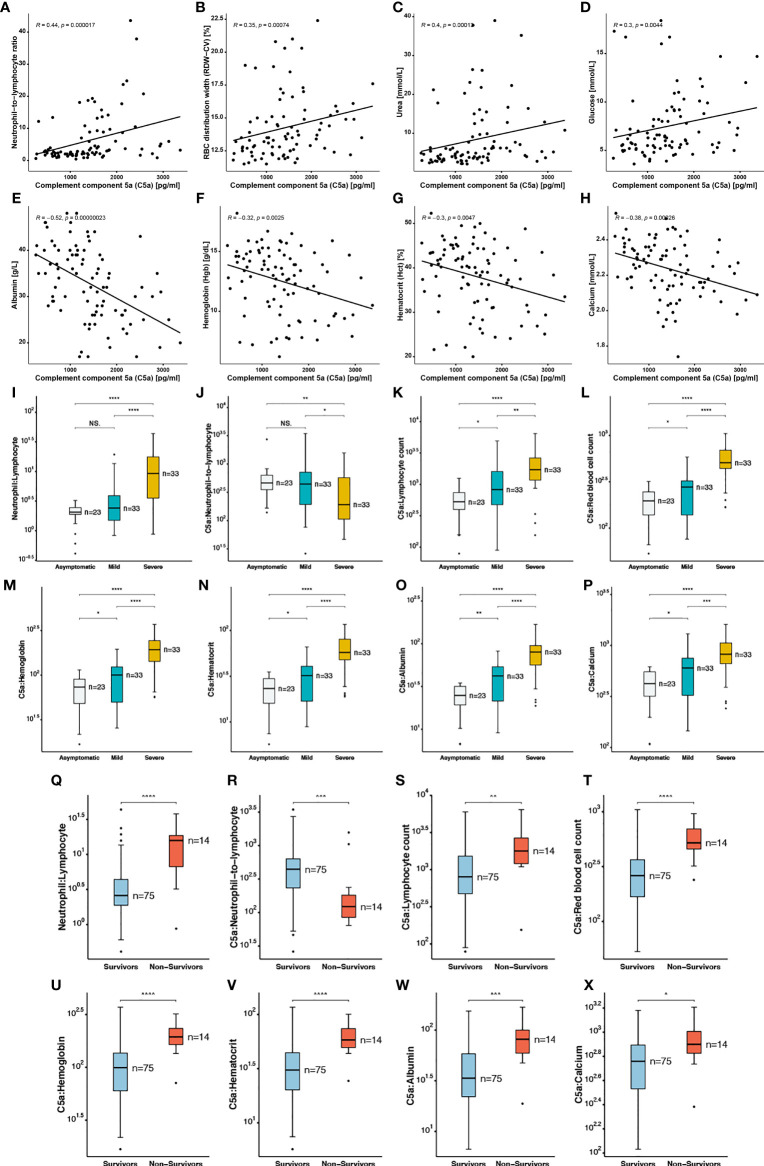
Correlation of complement component 5a with blood indices in SARS-CoV2 infected patients **(A)** Neutrophil−to−lymphocyte ratio, **(B)** RBC distribution width, **(C)** Urea, **(D)** glucose, **(E)** albumin, **(F)** hemoglobin, **(G)** hematocrit, **(H)** calcium. Ratios of complement component 5a to blood indices including **(I, Q)** neutrophil: Lymphocyte, **(J, R)** C5a: neutrophil−to−lymphocyte, **(K, S)** C5a: lymphocyte count, **(L, T)** C5a: red blood cell count, **(M, U)** C5a: hemoglobin, **(N, V)** C5a: hematocrit, **(O, W)** C5a: albumin, **(P, X)** C5a: calcium in asymptomatic, mild and severe COVID-19 patients, and in survivors versus non-survivors. The following symbols were used to indicate statistical significance: NS, p > 0.05; *p ≤ 0.05; **p ≤ 0.01; ***p ≤ 0.001; ****p ≤ 0.0001.

Elevated levels of pro-inflammatory anaphylatoxin C5a has been reported in cases of sepsis involving renal impairment. Neutrophil-to-lymphocyte ratio (NLR) is also recognized as a predictive factor for disease severity in sepsis, a variety of malignancies, and recently for critical illness in patients with SARS-CoV-2 infection. In accordance with these studies, a higher NLR was observed in the severe group patients compared to mild and asymptomatic cases (**Figure 3I**). However, it has not yet been established whether there is an association between C5a and other haematological and serological parameters, and the resulting long-term outcomes in COVID-19 patients.

A significantly lower ratio of C5a to NLR (CNLR) was calculated in severe cases of COVID-19 compared to less severe patients. In addition, we report a statistically significant lower median of CNLR in the non-survivor group (**Figure 3R**). In order to further investigate the role of C5a in COVID-19 pathogenesis, we calculated the ratios of C5a to several blood indices and found that ratios of C5a to lymphocyte (**Figure 3K**), RBC (**Figure 3L**), Hgb (**Figure 3M**), Hct (**Figure 3N**), albumin (**Figure 3O**), and calcium (**Figure 3P**) were significantly higher in severe patients in comparison with patients with no or mild symptoms (**Figure 3** and **Supplementary Table 2**). A correlation matrix was plotted for all investigated covariates to analyse the relationship between each pair of variables in this dataset (**Supplementary Figure 4**). Moreover, Spearman’s Correlation analysis revealed that neutrophil-to-lymphocyte ratio (**Figure 3A**), red blood cell distribution width (RDW) (**Figure 3B**), urea (**Figure 3C**), and glucose (**Figure 3D**) were positively correlated with C5a levels. A mild correlation (R=0.44) observed between C5a and NLR. On the other hand C5a levels had an inverse correlation with albumin (**Figure 3E**), Hgb (**Figure 3F**), Hct (**Figure 3G**) and calcium (**Figure 3H**), with a mild inverse correlation (R=0.52) with albumin. Altogether, C5a might contribute to COVID-19 disease severity by exacerbating innate immune responses and renal and hepatic injury while playing a dual role in inflammation and thrombosis.

### C5a Is a Novel Predictive Marker For Mortality in COVID-19 Patients

**Figure 4:**
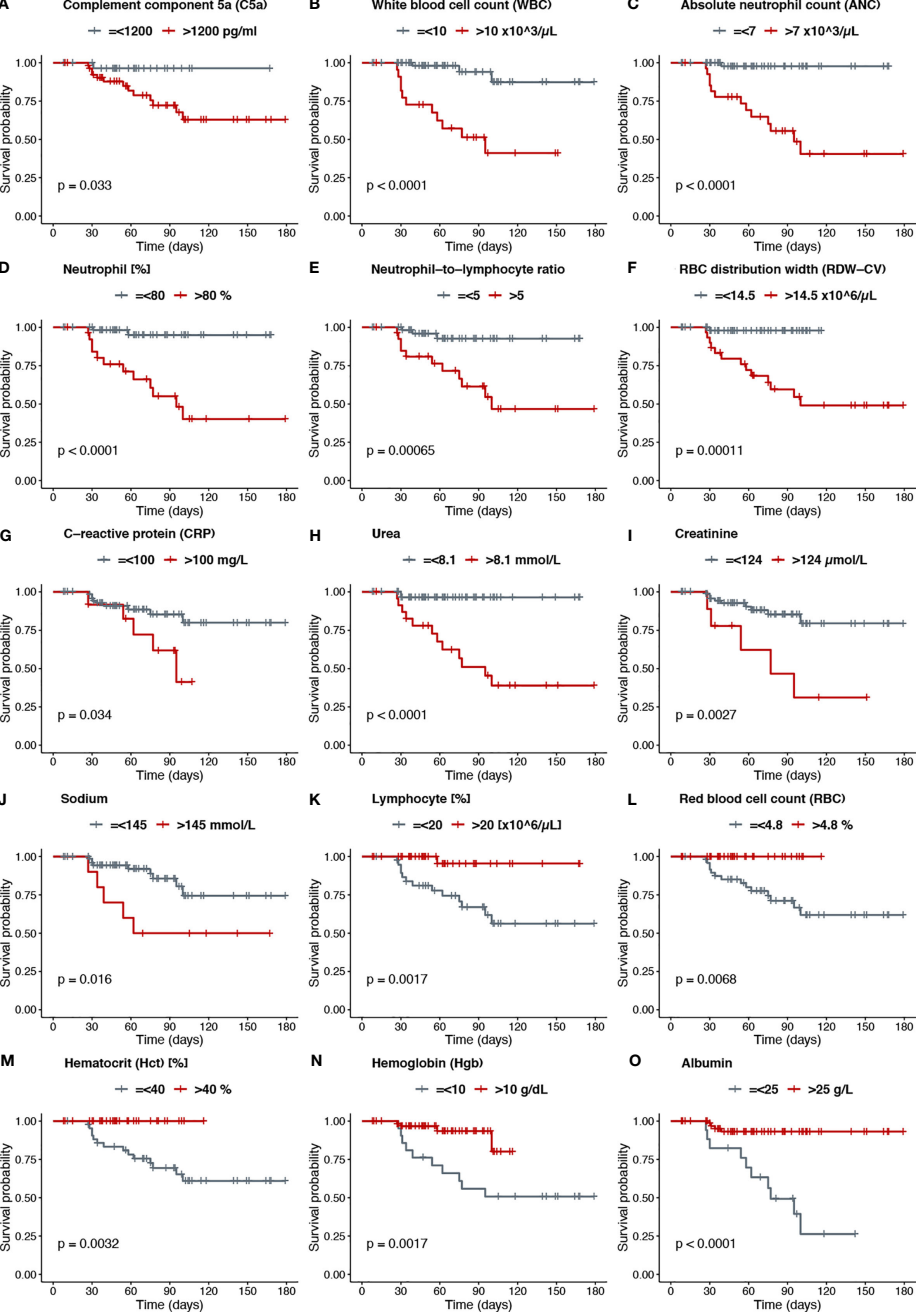
**(A)** Complement component 5a (C5a), **(B)** White blood cell count (WBC), **(C)** Absolute neutrophil count (ANC), **(D)** Neutrophil [%], **(E)** Neutrophil−to−lymphocyte ratio, **(F)** RBC distribution width (RDW−CV), **(G)** C−reactive protein (CRP), **(H)** Urea, **(I)** Creatinine, **(J)** Sodium, **(K)** Lymphocyte [%], **(L)** Red blood cell count (RBC), **(M)** Hematocrit (Hct) [%], **(N)** Hemoglobin (Hgb), **(O)** Albumin.

**Figure 5:**
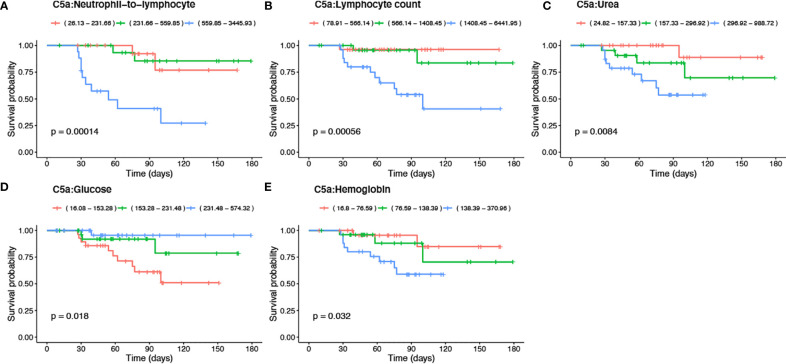
Kaplan-Meier survival analysis using tertiles of C5a-to blood indices including -NLR, -lymphocyte, -urea, -glucose and -Hgb ratios. The significance of the log-rank Mantel–Cox test of equality of survival distributions is shown as *p*-value. Ratios of C5a-to blood indices were stratified based on tertiles as indicated in the reference ranges and represented with orange, green, and blue lines.

To investigate mortality risk factors in COVID-19 patients, available clinical and laboratory parameters were stratified based on clinically relevant cut-offs using normal reference intervals. The difference between the time of admission or quarantine and the time of death or end of the survey was used to calculate Kaplan-Meier survival estimates. Increased levels of C5a in the plasma of severe cases, prompted an inclusion of C5a in survival analysis, along with already described risk factors. The survival function graph demonstrated that levels of C5a higher than 1200 pg/ml adversely affect short-term survival in COVID-19 patients (p=0.033) (**Figure 4A**). Notably, the two functions are closer together in the first 30-40 days of follow-up, but thereafter have a widening gap, suggesting that high levels of C5a is more detrimental later during follow-up than it is early on. To further evaluate the relationship between C5a and known prognostic markers with mortality status, ratios of C5a to other covariates were independently stratified into tertiles and assessed as predictors of survival. We found that ratios of C5a-to-NLR (**Figure 5A**), C5a-to-lymphocyte (**Figure 5B**), C5a-to-urea (**Figure 5C**), C5a-to-glucose (**Figure 5D**), and C5a-to-Hgb (**Figure 5E**) are good predictors of mortality in SARS-CoV-2 infection. Additionally, analysing CBC variables demonstrated that patients with high cut-off for WBC (>10x10^3^/μL) (**Figure 4B**), ANC (>7x103/μL) (**Figure 4C**), neutrophil percentage (>80%) (**Figure 4D**), neutrophil-to-lymphocyte ratio (>5) (**Figure 4E**), RDW-CV (>14.5%) (**Figure 4F**) exhibited a higher risk for mortality. Furthermore, patients with a low probability of survival showed a higher level of inflammatory marker CRP >100 mg/L (**Figure 4G**), as well as, urea >8.1mmol/L (**Figure 4H**), and creatinine >124 μmol/L (**Figure 4I**). Patients with high sodium levels (>145 mmol/L) tended to have a higher risk of death (**Figure 4J**). Additionally, the study of the survival time using other potential prognostic blood indices revealed several independent risk factors that are associated with fatal outcome including low levels of lymphocyte (=<20%) (**Figure 4K**), albumin (=<25 g/L) (**Figure 4O**), and red blood cell parameters including RBC (=<4.8x10^6^/µL) (**Figure 4L**), Hct (=<40%) (**Figure 4M**), and Hgb (=<10 g/dL) (**Figure 4N**) in SARS-CoV-2 infected patients.

### Validation of Severity and Mortality Models: CAL Is a New Predictive Model of COVID-19 Severity

**Figure 6:**
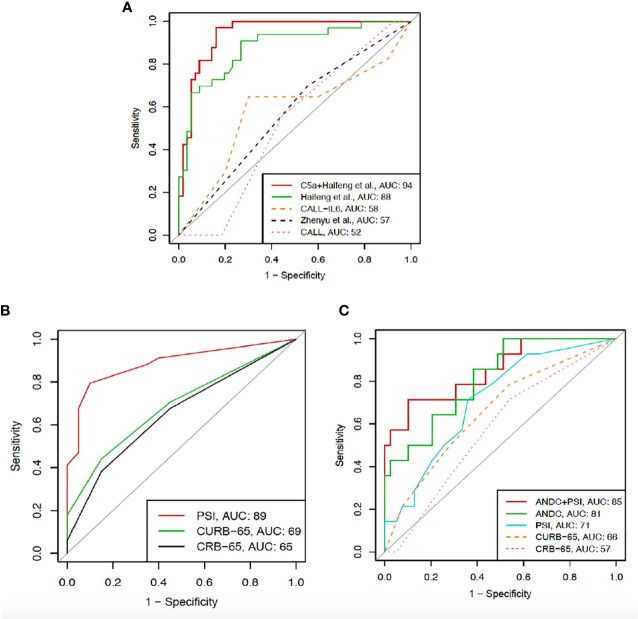
ROC curves showing the comparison between CAL (C5a, albumin, and lymphocyte count) and previously described severity models **(A)** including Haifeng et al. (lymphocyte count, albumin), CALL‐interleukin‐6 (IL‐6), Zhenyu et al. (age, albumin, comorbidity, CRP), and CALL (comorbidity, age, lymphocyte count, lactate dehydrogenase). **(B)** ROC curves for CRB-65, CURB-65, and PSI in predicting ICU admission. **(C)** ROC curves for CRB-65, CURB-65, and PSI alone, ANDC alone and PSI with ANDC combined in predicting mortality.

Based on the original formulas, we were able to calculate severity scores for the CALL, CALL-IL6, Haifeng et al. and Zhenyu et al. models in 89 (99%) patients. Comparing the AUROCs for severe COVID-19, only the model by Haifeng et al. was statistically significant with AUC 0.88 (95% CI 0.80-0.95) (32). Performance of severity models can be found in **Figure 6A**. Multivariable logistic regression considering the Haifeng et al. model variables (lymphocyte count and albumin levels) along with C5a levels as predictors of severe COVID-19, showed that only the latter two variables were significant (OR 0.707, 95% CI 0.5817–0.815) and (OR 1.001, 95% CI 1.0002–1.003), respectively. Thus, we tested the hypothesis that adding C5a to albumin, and lymphocyte and referring to it as the CAL would result in a predictive model with improved discriminative ability. Using the Delong approach to compare AUCs (42), the CAL model performed better than the original version with (AUC 0.94 vs. 0.88, with difference between areas 0.06, p= 0.04) (**Figure 6A**), but had a lower overall calibration (**Supplementary Figure 5**). Using the original formulas or points, we were able to calculate mortality scores for the CURB-65, CRB-65, PSI and ANDC models in 53 (60%) patients. Only the ANDC and PSI models were significant, with AUCs 0.81 (95% CI 0.69-0.94) and 0.71 (95% CI 0.56-0.86), respectively. Therefore, we tested the performance of both PSI and ANDC combined, which showed better accuracy, AUC 0.85 (95% CI 0.73-0.98) (**Figure 6B**). The latter model showed better calibration than the ANDC alone (**Supplementary Figure 5**). Furthermore, using the original formulas or points, we tested predicting ICU admission using the CURB-65, CRB-65, and PSI scores in 53 (60%) patients. Only the PSI excellent performance in predicting ICU admission AUC 0.88 (95% CI 0.79-0.97) (**Figure 6B**) with acceptable calibration (**Supplementary Figure 5**).

## Discussion

Severe SARS-CoV-2 infection confers a hypercoagulable state along with a robust inflammatory response (7, 8). Evidence gathered during post-mortem examination of COVID-19 patients implicates thrombosis as a major cause of death. Several studies demonstrated that an exaggerated anti-viral inflammatory response can lead to endothelial dysfunction/activation and a procoagulant state, known as thrombotic microangiopathy (TMA) (43). TMA results in diminished blood flow leading to tissue ischemia and oxidative injury, culminating in multiorgan failure reported in COVID-19 patients. In agreement, high incidence of venous thromboembolism (VTE) has been shown to correlate with disease severity and mortality in hospitalized COVID-19 patients (44). Accordingly, pulmonary intravascular coagulation observed in the lungs of COVID-19 patients can compromise other organs including the heart and kidneys, leading to multiorgan failure and death (9, 45–47). Recent studies demonstrated the role of SARS-CoV-2 in complement activation (48). The SARS-CoV-2 N protein was reported to bind mannan-binding lectin-associated protease (MASP-2) and activate the lectin pathway, initiating the complement cascade (49). Complement activation products were also detected on circulating erythrocytes in hospitalized COVID-19 patients (50). Particularly, serum levels of anaphylatoxin C5a are significantly increased in COVID-19 patients (51, 52). Furthermore, antibodies against SARS-CoV-2 may also contribute to the activation of the classical and alternative pathways of complement, sustaining high levels of C5a in severe COVID-19 cases (53). In another study using immunohistochemistry staining and single-cell RNA-Seq, high expression level of C5aR1 across inflammatory cells was detected in the lungs of patients infected with SARS-CoV-2 (38, 54, 55). Increased plasma levels of complement terminal complex soluble C5b-9 (sC5b-9) and activated C5a correlated with disease severity. Even more, elevated levels of C5a were found in patients requiring continuous positive airway pressure or mechanical ventilation (52). In addition, histological studies in COVID-19 patients have shown deposition of viral spike glycoprotein, complement split product C4d, and sC5b-9 in the interalveolar septa and microvessels (56). Similarly, Jiang et al. detected high concentrations of C5a and sC5b-9 in the sera and lung tissue in a mouse model of MERS (51). Our study is in alignment with previous research demonstrating a crucial role of C5a in the crosstalk between inflammation and thrombosis (38, 48–50, 52, 54, 56). We demonstrated increased levels of C5a in the plasma of our multi-ethnic cohort of COVID-19 patients, which was proportional to disease severity. Increases in C5a levels were also proportional to the levels of fibrin degradation product, D-dimer, known to be elevated in VTE and disseminated intravascular coagulation. Additionally, we found poor survival outcomes in COVID-19 patients with C5a levels higher than 1200 pg/ml. In our data, both C5a levels and neutrophil count increased proportionally with disease severity. The complement component C5a is a potent chemoattractant, which activates neutrophils and recruits them to the site of inflammation (57). The activation of C5a results in neutrophil degranulation and tissue factor expression, resulting in a prothrombotic state by triggering the extrinsic coagulation pathway (23). In this line, we found a positive correlation between C5a levels and the number of circulating neutrophils in the blood, in addition to a lower ratio of C5a to NLR (CNLR) in the severe cases of COVID-19. Here we propose “CAL” as a novel prognostic model of COVID-19 severity with an enhanced predictive capacity.

Similarly, previous reports have detected low serum albumin in patients with severe COVID-19, which was linked to thrombotic events (58, 59). In this study, an association of hypoalbuminemia with patient mortality was identified. A sustained stress on the liver leading to the production of APPs and clotting factors diverting the resources might result in a diminished synthesis of albumin, further aggravating the hemodynamic status. While the insult on the renal system led to increased levels of circulating urea and creatinine promoting multi-organ damage. The role of proteinuria as a cause of hypoalbuminemia needs further exploration. We further attempted to validate several COVID-19 severity models created to aid in clinical decision-making, but exhibit limited overall performance. These models are associated with a risk of bias and overfitting and are not well-reported (60). Public sharing of anonymized raw data that has been published from COVID-19 studies is necessary to develop and validate better models in large multi-centred settings (60). We acknowledge that the current study has limited sample size and the model needs further validation in diverse ethnic backgrounds with larger cohort studies. Moreover, the CAL model might not be easy to adopt in low-income countries due to financial constraints associated with lab investigations in the underdeveloped countries.

**Figure 7:**
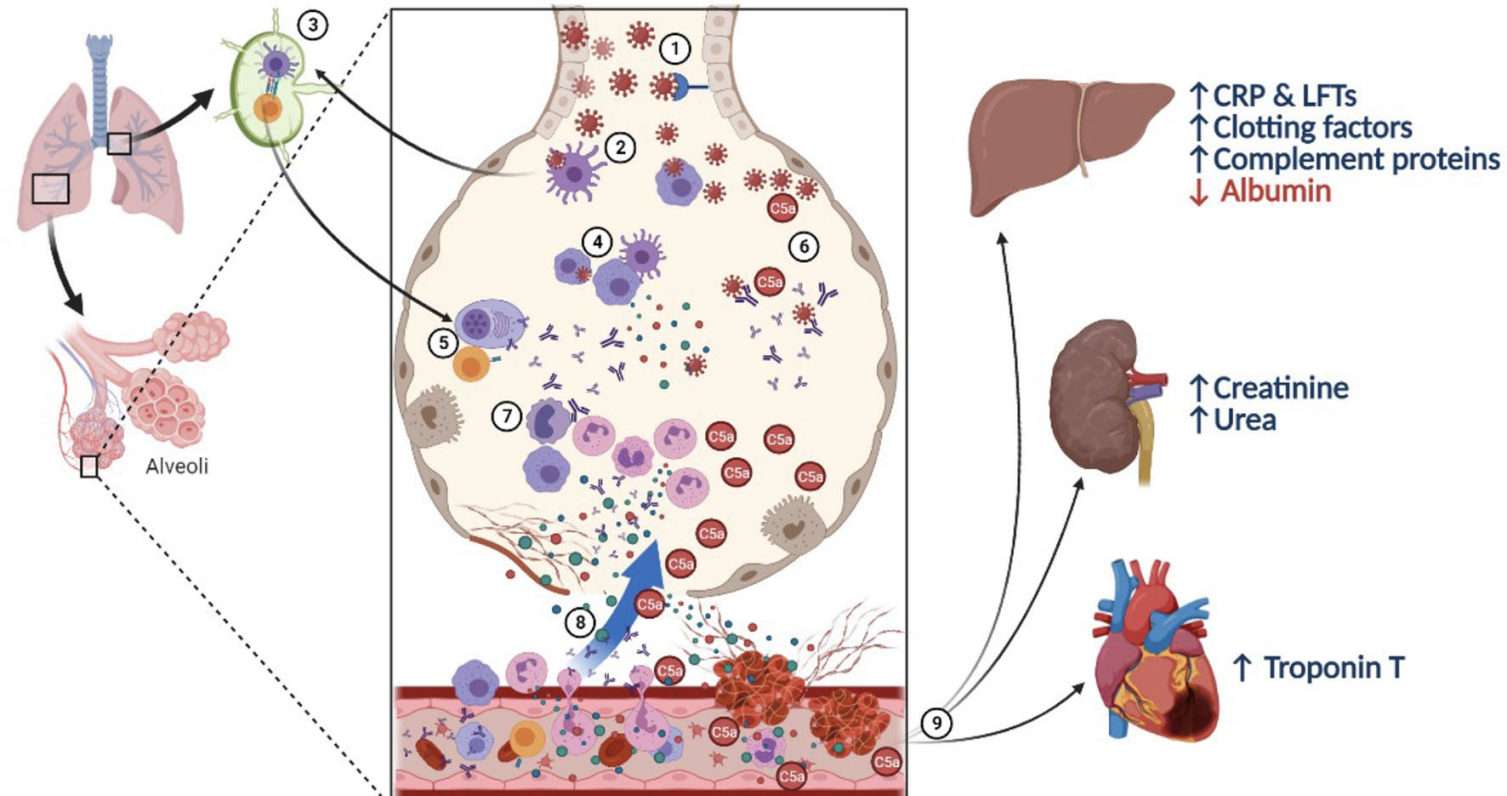
A proposed mechanistic role of complement C5a in lung injury during SARS-CoV-2 infection. (1) SARS-CoV-2 virus infects respiratory epithelial cells by binding to the angiotensin-converting enzyme 2 (ACE-2) receptor, followed by viral shedding. (2) Tissue resident macrophages and dendritic cells take up the virus or virus-infected cells. (3) They carry out antigen presentation in the hilar lymph nodes activating virus specific T and B cells. (4) Innate immune cells in the pulmonary microenvironment release cytokines to recruit immune cells to the site of infection. (5) T lymphocytes help B cells and plasma cells release antibodies into circulation and in the pulmonary microenvironment. (6) Meanwhile, complement activation can take place directly *via* lectin pathway or *via* alternative and classical pathways in the presence of antigen-antibody complexes. (7) C5a acts as a potent chemotactic agent recruiting innate immune cells, including neutrophils which further activates the immune system leading to cytokine storm. (8) Damaged alveoli and leaky blood vessels promote local clotting with C5a and IL-6, stimulating platelet activation and release of pro-inflammatory cytokines like IL-6 and C5a into circulation. (9) Systemic action of inflammatory mediators and micro-thrombi cause general tissue hypoxia along with stress on the liver leading to synthesise of acute phase reactants, and proteins from the complement and clotting cascade on the expense of albumin, with sequestration of ferritin, thereby elevating liver enzymes and promoting the pro-coagulant state. The inflammatory mediators, including immune complexes and C5a cause renal injury and cardiac stress.

In summary, high C5a complement protein and APPs, hypoalbuminemia, and renal insufficiency collectively have an adverse outcome on survival of COVID-19 patients. Altogether, we conclude that patients with an abnormally low level of albumin and lymphocytes and a high number of neutrophils, in addition to anaemic state characterized by low RBC, haemoglobin, and haematocrit counts are at a higher mortality risk. Patients who did not survive had elevated levels of inflammatory (CRP, and procalcitonin) and prothrombotic mediators (C5a, D-Dimer, INR, MPV, and PDW, prothrombin time, and partial thromboplastin time). Based on these results, we propose a mechanistic role of C5a in the pathogenesis and severity of COVID-19, highlighting its crosstalk between inflammation and thrombosis (**Figure 7**). Lastly, this study identifies biomarkers of COVID-19 severity that can potentially assist clinicians in early recognition of patients at risk for critical complications and mortality and in developing new management strategies.

## Data Availability Statement

The raw data supporting the conclusions of this article will be made available by the authors, without undue reservation.

## Ethics Statement

The studies involving human participants were reviewed and approved by Medical Research Center Institutional Review Board MRC IRB, Hamad Medical Corporation. The patients/participants provided their written informed consent to participate in this study.

## Author Contributions

FC, GG, and MS: Data curation, methodology, formal analysis, and writing the original draft. IA and AD: methodology, investigation, data cleansing, formal analysis, and second draft. MYS, AN-E, MD, MA, MM, AZ, ALA, and AA: Sample acquisition, investigation. FM and AP: resources, investigation, and experimental design. EE and RS: Resources, supervision, experimental design. FC, GG, and MS: Experimental design, conceptualization, resources, supervision, funding acquisition, validation, investigation, visualization, project administration, and writing — review and editing. All authors contributed to the article and approved the submitted version.

## Funding

This work was supported by grant QUST-1-CMED-2021-2 awarded at Qatar university to Farhan Cyprian.

## Conflict of Interest

The authors declare that the research was conducted in the absence of any commercial or financial relationships that could be construed as a potential conflict of interest.

## Publisher’s Note

All claims expressed in this article are solely those of the authors and do not necessarily represent those of their affiliated organizations, or those of the publisher, the editors and the reviewers. Any product that may be evaluated in this article, or claim that may be made by its manufacturer, is not guaranteed or endorsed by the publisher.

## Supplementary Material

Supplementary Table 1 Demographic and clinical characteristics based on survival and hospital death in COVID-19 patients.

Supplementary Table 2 Ratios of complement component 5a to blood indices in asymptomatic, mild, and severe COVID-19 patients.

Supplementary Figure 1 Clinical features in COVID-19 patients across asymptomatic, mild, and severe cases **(A)** diastolic blood pressure, **(B)** body Mass Index (BMI), and **(C)** COVID−19 Average CT.

Supplementary Figure 2 Complete blood count parameters in COVID-19 patients who survived and those who did not survive **(A)** white blood cell count, **(B)** absolute neutrophil count, **(C)** neutrophil (%), **(D)** mean corpuscular volume, **(E)** ferritin, **(F)** red blood cell distribution width, **(G)** lymphocyte count, **(H)** lymphocyte (%), **(I)** monocyte (%), **(J)** red blood cell count, **(K)** hematocrit, **(L)** hemoglobin.

Supplementary Figure 3 Coagulation and inflammatory markers in COVID-19 patients who survived and those who did not survive **(A)** complement component 5a, **(B)** mean platelet volume, **(C)** platelet distribution width, **(D)** partial thromboplastin time, **(E)** D−dimer, **(F)** C−reactive protein, **(G)** procalcitonin, **(H)** adjusted calcium, (I) urea, (J) total protein, **(K)** albumin, **(L)** calcium, **(M)** high−sensitivity Troponin−T, **(N)** alkaline phosphatase, **(O)** alanine aminotransferase, **(P)** aspartate aminotransferase.

Supplementary Figure 4 Spearman correlation matrix for investigated covariates showing R values. Colour scale ranges from blue (r = −1) to white (r = 0) to red (r = 1).

Supplementary Figure 5
**(A)** Comparison of calibration of the original model by Haifeng et al. (left) with added C5a (right) in predicting severity. **(B)** Calibration of the ANDC+PSI model (top), ANDC model (bottom left) and the PSI score (bottom right) in predicting COVID-19 mortality. **(C)** Calibration of the PSI score in predicting ICU admission.
