# Supplementary material for: Complement C5a and Clinical Markers as Predictors of COVID-19 Disease Severity and Mortality in a Multi-Ethnic Population
Source: Front Immunol. 2021 Dec 13;12:707159. doi: 10.3389/fimmu.2021.707159 (PMC8710484; doi:10.3389/fimmu.2021.707159)
Supplement: Supplementary Table 1 — Demographic and clinical characteristics based on survival and hospital death in COVID-19 patients. [file DataSheet_1.docx]

**Supplementary Table 1** Demographic and clinical characteristics based on survival and hospital death in COVID-19 patients.

| Characteristic | N | Total  N = 89^1^ | Survivor  N = 75^1^ | Non-survivor  N = 14^1^ | *p*-value^2^ |
| --- | --- | --- | --- | --- | --- |
| **Severity** | 89 |  |  |  | **<0.001** |
| Asymptomatic |  |  | 23 (100%) | 0 (0%) |  |
| Mild |  |  | 33 (100%) | 0 (0%) |  |
| Severe |  |  | 19 (58%) | 14 (42%) |  |
| **Gender (Male) [%]** | 89 |  | 67 (83%) | 14 (17%) | 0.3 |
| **At least 1 Comorbidity** | 89 |  | 47 (85%) | 8 (15%) | >0.9 |
| **At least 2 Comorbidities** | 89 |  | 27 (84%) | 5 (16%) | >0.9 |
| **At least 3 Comorbidities** | 89 |  | 16 (89%) | 2 (11%) | 0.7 |
| **Hospitilization duration** | 89 | 7 (0-32) | 6 (0-16) | 48 (31-73) | **<0.001** |
| **Age [years]** | 89 | 48 (42-56) | 46 (41-54) | 57 (52-60) | **<0.001** |
| **COVID-19 Average CT** | 78 | 25.6 (18.7-29.5) | 25.7 (18.7-29.6) | 22.1 (19.1-28.1) | 0.6 |
| **Diastolic blood pressure [mmHg]** | 89 | 75 (64-83) | 75 (68-84) | 60 (51-72) | **0.006** |
| **Body Mass Index (BMI) [kg/m^2^]** | 68 | 28.1 (24.8-30.4) | 28.6 (25.0-30.5) | 27.2 (24.3-29.7) | 0.6 |
| **Nutritional status** | 68 |  |  |  | 0.8 |
| Normal weight |  |  | 13 (72%) | 5 (28%) |  |
| Obesity class I |  |  | 11 (85%) | 2 (15%) |  |
| Obesity class II |  |  | 2 (67%) | 1 (33%) |  |
| Obesity class III |  |  | 2 (100%) | 0 (0%) |  |
| Pre-obesity |  |  | 25 (81%) | 6 (19%) |  |
| Underweight |  |  | 1 (100%) | 0 (0%) |  |
| **Glucose [mmol/L]** | 89 | 6.3 (5.3-8.8) | 6.2 (5.2-8.3) | 6.8 (6.0-9.3) | 0.2 |
| **White blood cell count (WBC) [x10^3^/µL]** | 89 | 7.1 (4.9-10.3) | 6.6 (4.8-8.4) | 12.9 (11.5-18.6) | **<0.001** |
| **Lymphocyte count [x10^3^/µL]** | 89 | 1.40 (1.00-1.90) | 1.60 (1.15-1.95) | 0.95 (0.70-1.08) | **0.007** |
| **Absolute neutrophil count (ANC) [x10^3^/µL]** | 89 | 4.6 (3.1-7.8) | 4.3 (2.8-6.3) | 10.7 (9.4-12.9) | **<0.001** |
| **Neutrophil-to-lymphocyte ratio** | 89 | 2.8 (2.0-8.7) | 2.6 (1.9-4.4) | 15.9 (6.9-18.6) | **<0.001** |
| **Monocyte count[x10^3^/µL]** | 89 | 0.60 (0.40-0.80) | 0.60 (0.35-0.80) | 0.70 (0.40-0.80) | 0.3 |
| **Eosinophil count[x10^3^/µL]** | 89 | 0.00 (0.00-0.10) | 0.00 (0.00-0.20) | 0.00 (0.00-0.10) | 0.14 |
| **Basophil count[x10^3^/µL]** | 89 | 0.030 (0.010-0.040) | 0.030 (0.010-0.040) | 0.035 (0.013-0.057) | 0.4 |
| **Lymphocyte [%]** | 89 | 23 (9-30) | 25 (17-31) | 5 (4-8) | **<0.001** |
| **Neutrophil [%]** | 89 | 68 (58-83) | 65 (55-75) | 88 (85-91) | **<0.001** |
| **Monocyte [%]** | 89 | 7.7 (5.2-9.3) | 8.0 (5.8-9.6) | 5.0 (3.6-6.9) | **0.004** |
| **Eosinophil [%]** | 89 | 0.70 (0.00-1.90) | 0.90 (0.10-2.30) | 0.20 (0.00-0.50) | **0.008** |
| **Basophil [%]** | 89 | 0.30 (0.20-0.50) | 0.40 (0.20-0.55) | 0.25 (0.10-0.38) | **0.046** |
| **Red blood cell count (RBC) [x10^6^/µL]** | 89 | 4.70 (3.80-5.30) | 4.90 (4.20-5.35) | 3.20 (2.68-3.48) | **<0.001** |
| **Hematocrit (Hct) [%]** | 89 | 40 (33-44) | 41 (37-44) | 28 (25-32) | **<0.001** |
| **Hemoglobin (Hgb) [g/dL]** | 89 | 13.00 (10.40-14.70) | 13.60 (11.90-14.90) | 8.95 (7.73-10.25) | **<0.001** |
| **Hemoglobin A1C (HbA1C) [%]** | 47 | 6.20 (5.60-7.55) | 6.20 (5.57-7.53) | 6.20 (6.05-7.15) | 0.6 |
| **Ferritin [µg/L]** | 62 | 661 (286-1,151) | 611 (253-1,090) | 1,542 (459-4,584) | **0.017** |
| **Mean corpuscular volume (MCV) [fL]** | 89 | 86 (82-91) | 86 (82-89) | 90 (87-95) | **0.012** |
| **Mean cell hemoglobin (MCH) [pg]** | 89 | 28.60 (26.70-30.00) | 28.60 (26.50-30.00) | 28.85 (26.72-29.78) | 0.9 |
| **Mean corpuscular hemoglobin concentration (MCHC) [g/dL]** | 89 | 33.10 (31.60-33.90) | 33.30 (31.90-34.05) | 32.25 (31.08-33.00) | **0.029** |
| **Red blood cell distribution width (RDW-CV) [%]** | 89 | 13.50 (12.50-15.10) | 13.20 (12.35-14.40) | 17.05 (15.27-18.27) | **<0.001** |
| **Platelet [× 10^9^/L]** | 85 | 246 (194-326) | 262 (201-332) | 165 (151-231) | **0.013** |
| **Mean platelet volume (MPV) [fl]** | 86 | 10.55 (9.70-11.38) | 10.50 (9.55-11.10) | 12.30 (11.10-12.95) | **<0.001** |
| **Platelet distribution width (PDW) [fl]** | 56 | 13.5 (11.5-15.7) | 12.9 (10.9-15.5) | 16.0 (13.1-20.4) | **0.010** |
| **Prothrombin time (PT) [second]** | 55 | 12.10 (11.35-13.20) | 12.10 (11.20-12.60) | 12.95 (11.80-15.22) | 0.12 |
| **International Normalized Ratio (INR)** | 55 | 1.00 (1.00-1.10) | 1.00 (1.00-1.10) | 1.05 (1.00-1.28) | 0.3 |
| **D-Dimer [mg/L FEU]** | 64 | 0.87 (0.37-2.40) | 0.52 (0.30-1.90) | 4.15 (1.49-5.67) | **<0.001** |
| **Fibrinogen [g/L]** | 40 | 4.15 (3.00-5.67) | 4.65 (3.73-5.83) | 3.10 (2.40-4.62) | **0.038** |
| **Partial thromboplastin time (APTT) [second]** | 55 | 31 (28-37) | 31 (28-34) | 40 (32-47) | **0.006** |
| **Complement component 5a (C5a) [pg/ml]** | 89 | 1,383 (929-1,786) | 1,289 (775-1,690) | 1,670 (1,375-2,191) | **0.035** |
| **C-reactive protein (CRP) [mg/L]** | 89 | 16 (5-58) | 13 (5-40) | 74 (21-158) | **0.004** |
| **Interleukin-6 (IL-6) [pg/mL]** | 52 | 32 (12-57) | 24 (8-53) | 43 (32-61) | 0.076 |
| **High-sensitivity Troponin-T [ng/mL]** | 40 | 26 (8-108) | 10 (7-33) | 84 (32-158) | **0.004** |
| **Lactic acid [mmol/L]** | 35 | 1.70 (1.10-2.20) | 1.37 (1.00-1.90) | 2.30 (1.80-2.70) | **0.010** |
| **Uric acid [µmol/L]** | 51 | 305 (266-384) | 323 (277-391) | 260 (146-292) | **0.028** |
| **Bicarbonate [mmol/L]** | 89 | 25.0 (23.0-28.0) | 25.0 (23.0-27.0) | 27.0 (24.0-31.8) | 0.2 |
| **Sodium [mmol/L]** | 89 | 138.0 (136.0-141.0) | 138.0 (136.0-140.0) | 144.0 (136.2-149.0) | 0.063 |
| **Potassium [mmol/L]** | 85 | 4.40 (4.00-4.70) | 4.30 (4.00-4.70) | 4.55 (4.32-4.85) | 0.2 |
| **Chloride [mmol/L]** | 89 | 102.0 (99.0-104.0) | 101.5 (99.0-104.0) | 103.0 (95.8-107.8) | 0.8 |
| **Phosphorus [mmol/L]** | 46 | 1.14 (1.00-1.31) | 1.13 (1.02-1.26) | 1.27 (0.97-1.47) | 0.2 |
| **Magnesium [mmol/L]** | 60 | 0.89 (0.83-0.96) | 0.87 (0.83-0.96) | 0.96 (0.87-1.05) | **0.044** |
| **Total Protein [g/L]** | 80 | 71 (64-77) | 71 (68-77) | 56 (54-74) | **0.012** |
| **Albumin [g/L]** | 89 | 32 (27-39) | 35 (30-40) | 22 (19-26) | **<0.001** |
| **Bilirubin [mg/dL]** | 80 | 8 (5-13) | 8 (5-11) | 16 (7-26) | **0.017** |
| **Urea [mmol/L]** | 89 | 5 (4-10) | 4 (3-6) | 21 (15-26) | **<0.001** |
| **Creatinine [µmol/L]** | 89 | 78 (63-92) | 76 (62-90) | 90 (65-226) | 0.15 |
| **Creatine kinase (CK) [U/L]** | 45 | 103 (58-245) | 74 (56-182) | 204 (67-282) | 0.2 |
| **Alkaline phosphatase (ALP) [U/L]** | 80 | 88 (63-110) | 80 (61-99) | 161 (129-302) | **<0.001** |
| **Alanine aminotransferase (ALT) [U/L]** | 77 | 31 (20-64) | 30 (19-52) | 73 (32-136) | **0.009** |
| **Aspartate aminotransferase (AST) [U/L]** | 72 | 28 (23-50) | 26 (20-43) | 70 (35-120) | **<0.001** |
| **Lactate dehydrogenase (LDH) [U/L]** | 53 | 347 (249-462) | 322 (228-408) | 576 (382-1,090) | **<0.001** |
| **Low-density lipoprotein (LDL) [mmol/L]** | 16 | 2.85 (2.28-3.70) | 2.85 (2.28-3.70) | NA (NA-NA) |  |
| **Triglyceride [mmol/L]** | 52 | 1.80 (1.35-2.62) | 1.70 (1.20-2.20) | 2.65 (1.88-3.55) | **0.025** |
| **Vitamin D [ng/mL]** | 56 | 18 (13-23) | 18 (13-23) | 16 (14-29) | 0.7 |
| **Calcium [mmol/L]** | 89 | 2.25 (2.14-2.35) | 2.29 (2.17-2.36) | 2.12 (2.00-2.18) | **<0.001** |
| **Adjusted calcium [mmol/L]** | 89 | 2.39 (2.30-2.46) | 2.36 (2.30-2.46) | 2.46 (2.41-2.56) | **0.019** |
| **Procalcitonin [ng/mL]** | 47 | 0.27 (0.10-0.55) | 0.15 (0.06-0.31) | 0.86 (0.57-5.06) | **<0.001** |
| ^1^Statistics presented: n (%); Median (25%-75%) | | | | | |
| ^2^Statistical tests performed: Fisher's exact test; chi-square test of independence; Kruskal-Wallis test. Bold p-values are those below the threshold of 0.05. | | | | | |

**Supplementary Table 2** Ratios of complement component 5a to blood indices in asymptomatic, mild, and severe COVID-19 patients.

| Characteristic | N | Total  N = 89^1^ | Asymptomatic  N = 23^1^ | Mild  N = 33^1^ | Severe  N = 33^1^ | p-value^2^ |
| --- | --- | --- | --- | --- | --- | --- |
| **C5a : Neutrophil-to-lymphocyte ratio** | 89 | 409 (160-611) | 464 (353-632) | 444 (193-715) | 170 (90-527) | **0.002** |
| **C5a : Lymphocyte count** | 89 | 1,012 (503-1,687) | 528 (398-742) | 827 (475-1,604) | 1,854 (1,173-2,697) | **<0.001** |
| **C5a : Red blood cell count** | 89 | 284 (193-469) | 197 (139-247) | 278 (139-323) | 508 (440-694) | **<0.001** |
| **C5a : Hematocrit** | 89 | 35 (21-54) | 23 (16-30) | 32 (17-41) | 58 (48-80) | **<0.001** |
| **C5a : Hemoglobin** | 89 | 110 (65-166) | 73 (48-90) | 100 (50-124) | 193 (143-244) | **<0.001** |
| **C5a : Albumin** | 89 | 43 (23-64) | 25 (19-32) | 42 (21-54) | 80 (56-95) | **<0.001** |
| **C5a : Calcium** | 89 | 603 (394-812) | 423 (318-552) | 602 (325-751) | 821 (664-1,059) | **<0.001** |
| ^1^Statistics presented: n (%); Median (25%-75%) | | | | | | |
| ^2^Statistical test performed: Kruskal-Wallis test. Bold p-values are those below the threshold of 0.05. | | | | | | |


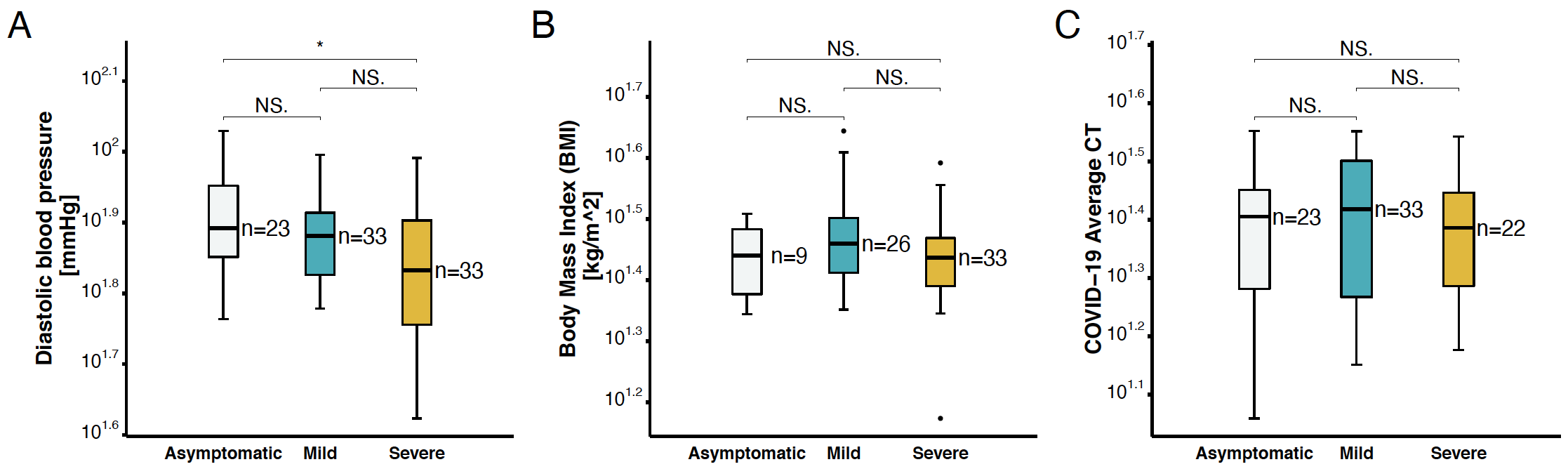


**Supplementary Figure 1**

**Supplementary Figure 2**

Supplementary Figure 3

**Supplementary Figure 4**


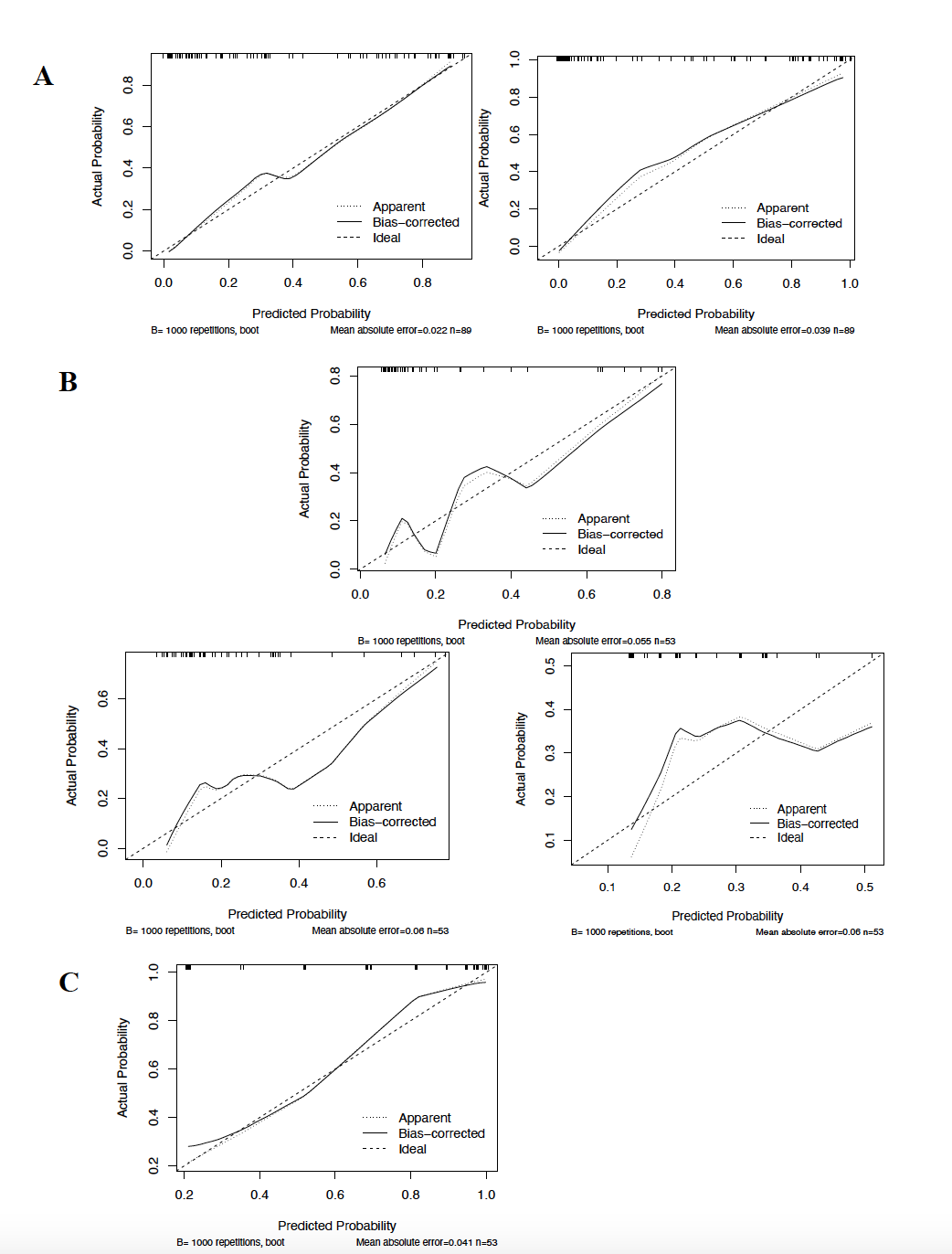


**Supplementary Figure 5**

**Supplementary Figure 1 Clinical features in COVID-19 patients across asymptomatic, mild, and severe cases** (A) diastolic blood pressure, (B) body Mass Index (BMI), and (C) COVID−19 Average CT.

**Supplementary Figure 2** **Complete blood count parameters in COVID-19 patients who survived and those who did not survive** (A) white blood cell count, (B) absolute neutrophil count, (C) neutrophil (%), (D) mean corpuscular volume, (E) ferritin, (F) red blood cell distribution width, (G) lymphocyte count, (H) lymphocyte (%), (I) monocyte (%), (J) red blood cell count, (K) hematocrit, (L) hemoglobin.

Supplementary Figure 3 **Coagulation and inflammatory markers in COVID-19 patients who survived and those who did not survive** (A) complement component 5a, (B) mean platelet volume, (C) platelet distribution width, (D) partial thromboplastin time, (E) D−dimer, (F) C−reactive protein, (G) procalcitonin, (H) adjusted calcium, (I) urea, (J) total protein, (K) albumin, (L) calcium, (M) high−sensitivity Troponin−T, (N) alkaline phosphatase, (O) alanine aminotransferase, (P) aspartate aminotransferase.

**Supplementary Figure 4** Spearman correlation matrix for investigated covariates showing R values. Colour scale ranges from blue (r = −1) to white (r = 0) to red (r = 1).

**Supplementary Figure 5** (A) Comparison of calibration of the original model by Haifeng et al (left) with added C5a (right) in predicting severity. (B) Calibration of the ANDC+PSI model (top), ANDC model (bottom left) and the PSI score (bottom right) in predicting COVID-19 mortality. (C) Calibration of the PSI score in predicting ICU admission.
